# Supplementary material for: A Likelihood Approach for Real-Time Calibration of Stochastic Compartmental Epidemic Models
Source: PLoS Comput Biol. 2017 Jan 17;13(1):e1005257. doi: 10.1371/journal.pcbi.1005257 (PMC5240920; doi:10.1371/journal.pcbi.1005257)
Supplement: S1 File — (TAR.GZ) [file pcbi.1005257.s014.tar.gz › HSPH_Online-SI-Revision/Additions/IPoi10/n100-extreme-SEITR_noisy/n100-extreme-SEITR_noisy_BENCH2_table8.pdf]

1	newly 3 weeks spec	50.75	1219.48	{626., 1888.}	2302.93	1.33262	newly 3 weeks cumul	584.434	4852.72	{2429., 7384.}	730.328	0.873231	newly 5 weeks spec	42.6203	708.859	{359., 1127.}	1563.2	1.17102	newly 5 weeks cumul	687.444	6491.44	{3243., 9937.}	844.286	0.928135	newly 42 weeks cumul	3753.74	8704.45	{4383., 13 399.}	132.463	0.319736
2	newly 3 weeks spec	102.	536.047	{228., 996.}	425.536	0.637435	newly 3 weeks cumul	593.	2629.3	{1203., 5000.}	343.389	0.565543	newly 5 weeks spec	36.	217.781	{88., 397.}	504.948	0.691352	newly 5 weeks cumul	673.	3189.86	{1433., 6058.}	373.976	0.593421	newly 50 weeks cumul	712.	3577.91	{1600., 6742.}	402.515	0.618377
3	newly 3 weeks spec	169.	100.859	{43., 189.}	49.9875	0.325093	newly 3 weeks cumul	920.	573.648	{259., 992.}	48.1828	0.304449	newly 5 weeks spec	52.	33.6338	{13., 58.}	49.2416	0.308852	newly 5 weeks cumul	1072.	663.507	{303., 1156.}	48.4326	0.306623	newly 52 weeks cumul	1137.	707.465	{320., 1245.}	48.3717	0.30572
4	newly 3 weeks spec	137.	347.167	{141., 729.}	154.663	0.334758	newly 3 weeks cumul	790.	1731.64	{702., 3442.}	123.537	0.288741	newly 5 weeks spec	41.	143.347	{59., 267.}	249.627	0.46938	newly 5 weeks cumul	909.	2096.97	{830., 4117.}	134.042	0.305424	newly 50 weeks cumul	985.	2352.71	{939., 4612.}	141.589	0.317582
5	newly 3 weeks spec	34.	82.8852	{42., 145.}	144.069	0.340223	newly 3 weeks cumul	171.	501.213	{267., 873.}	193.107	0.419021	newly 5 weeks spec	11.	24.8197	{11., 41.}	130.999	0.316531	newly 5 weeks cumul	195.	570.77	{300., 989.}	192.703	0.418309	newly 52 weeks cumul	201.	600.393	{316., 1030.}	198.703	0.427049
6	newly 3 weeks spec	5.	106.523	{56., 185.}	2030.46	1.26576	newly 3 weeks cumul	60.	617.338	{346., 1024.}	928.897	0.953034	newly 5 weeks spec	2.	35.1692	{17., 61.}	1658.46	1.17382	newly 5 weeks cumul	63.	715.015	{393., 1204.}	1034.95	0.99517	newly 51 weeks cumul	64.	764.154	{412., 1290.}	1093.99	1.01656
7	newly 3 weeks spec	469.	768.234	{366., 1446.}	76.1827	0.202201	newly 3 weeks cumul	2142.	3168.88	{1526., 5822.}	65.6761	0.189333	newly 5 weeks spec	221.	433.531	{206., 821.}	100.622	0.23973	newly 5 weeks cumul	2717.	4179.78	{2030., 7703.}	69.3078	0.19325	newly 43 weeks cumul	3226.	5449.02	{2605., 10 088.}	79.1426	0.204938
8	newly 3 weeks spec	225.	423.292	{230., 672.}	90.4684	0.245459	newly 3 weeks cumul	1088.	2094.94	{1175., 3352.}	94.6734	0.253909	newly 5 weeks spec	80.	170.954	{88., 269.}	115.115	0.293511	newly 5 weeks cumul	1321.	2534.97	{1396., 4040.}	94.1315	0.252516	newly 50 weeks cumul	1472.	2841.75	{1545., 4530.}	95.1777	0.254369
9	newly 3 weeks spec	19.	78.2063	{40., 136.}	311.612	0.559887	newly 3 weeks cumul	167.	478.667	{249., 831.}	187.007	0.40446	newly 5 weeks spec	5.	23.3651	{10., 40.}	369.206	0.611411	newly 5 weeks cumul	179.	546.286	{280., 957.}	205.223	0.429875	newly 52 weeks cumul	188.	575.016	{291., 1008.}	205.876	0.430339
10	newly 3 weeks spec	613.	550.957	{209., 1162.}	53.453	0.265696	newly 3 weeks cumul	2661.	2478.29	{1020., 5301.}	53.682	0.257626	newly 5 weeks spec	260.	263.435	{99., 565.}	56.1929	0.248395	newly 5 weeks cumul	3324.	3120.	{1277., 6687.}	53.9999	0.257077	newly 48 weeks cumul	3949.	3699.35	{1502., 7958.}	54.0926	0.257902
11	newly 3 weeks spec	448.	726.899	{339., 1284.}	75.8799	0.20999	newly 3 weeks cumul	2137.	3225.74	{1536., 5624.}	68.6409	0.201387	newly 5 weeks spec	237.	363.478	{158., 639.}	70.6476	0.204917	newly 5 weeks cumul	2689.	4102.48	{1953., 7210.}	69.7398	0.202544	newly 47 weeks cumul	3221.	4935.12	{2320., 8651.}	70.0404	0.202404
12	newly 3 weeks spec	209.	644.809	{224., 1511.}	210.632	0.372685	newly 3 weeks cumul	1086.	2996.9	{1074., 7177.}	178.858	0.333202	newly 5 weeks spec	68.	287.368	{95., 701.}	322.599	0.498788	newly 5 weeks cumul	1294.	3713.21	{1326., 8882.}	189.251	0.346197	newly 48 weeks cumul	1442.	4290.1	{1531., 10 303.}	199.379	0.359013
13	newly 3 weeks spec	300.	320.808	{167., 553.}	40.3333	0.165106	newly 3 weeks cumul	1508.	1542.18	{792., 2667.}	38.001	0.162945	newly 5 weeks spec	131.	139.301	{72., 234.}	38.1261	0.15696	newly 5 weeks cumul	1818.	1894.81	{996., 3253.}	38.2951	0.160815	newly 49 weeks cumul	2109.	2163.77	{1131., 3680.}	38.0847	0.16253
14	newly 3 weeks spec	681.	847.043	{391., 1573.}	43.9145	0.156734	newly 3 weeks cumul	2981.	3416.33	{1655., 6235.}	38.4561	0.147299	newly 5 weeks spec	331.	494.362	{234., 937.}	60.8258	0.187265	newly 5 weeks cumul	3718.	4556.42	{2237., 8322.}	42.6624	0.153749	newly 42 weeks cumul	4474.	6092.96	{3019., 11 147.}	51.3842	0.169916
15	newly 3 weeks spec	238.	823.333	{321., 1270.}	245.938	0.44023	newly 3 weeks cumul	1233.	3345.6	{1370., 5048.}	172.419	0.344909	newly 5 weeks spec	100.	465.542	{178., 714.}	365.542	0.562401	newly 5 weeks cumul	1479.	4430.54	{1787., 6646.}	199.99	0.382563	newly 43 weeks cumul	1667.	5791.36	{2313., 8709.}	247.412	0.442427
16	newly 3 weeks spec	233.	607.957	{280., 1190.}	160.963	0.354109	newly 3 weeks cumul	1269.	2603.29	{1220., 5055.}	107.801	0.264064	newly 5 weeks spec	91.	313.029	{142., 614.}	243.988	0.474005	newly 5 weeks cumul	1497.	3349.35	{1517., 6571.}	124.913	0.294367	newly 46 weeks cumul	1647.	4143.96	{1907., 8098.}	151.626	0.340246
17	newly 3 weeks spec	1.	210.197	{89., 437.}	20 919.7	2.24524	newly 3 weeks cumul	62.	1213.2	{544., 2443.}	1856.77	1.21683	newly 5 weeks spec	0.1	67.5455	{25., 147.}	67 445.5	2.74174	newly 5 weeks cumul	64.	1401.36	{615., 2836.}	2089.63	1.26555	newly 52 weeks cumul	64.	1490.67	{654., 3028.}	2229.17	1.29237
18	newly 3 weeks spec	93.	223.939	{109., 396.}	142.229	0.325683	newly 3 weeks cumul	605.	1237.5	{579., 2224.}	107.721	0.2628	newly 5 weeks spec	53.	79.3333	{36., 160.}	67.3528	0.198996	newly 5 weeks cumul	722.	1452.06	{697., 2572.}	104.671	0.257303	newly 51 weeks cumul	795.	1566.92	{755., 2752.}	100.905	0.250038
19	newly 3 weeks spec	465.	1240.41	{529., 2000.}	167.415	0.32772	newly 3 weeks cumul	2284.	5112.17	{2329., 8256.}	125.732	0.27424	newly 5 weeks spec	217.	669.549	{269., 1062.}	209.158	0.376016	newly 5 weeks cumul	2812.	6693.82	{2994., 10 801.}	139.582	0.293017	newly 46 weeks cumul	3336.	8409.62	{3735., 13 500.}	153.283	0.310266
20	newly 3 weeks spec	75.	200.292	{101., 337.}	167.056	0.380393	newly 3 weeks cumul	401.	1103.06	{545., 1891.}	175.078	0.394087	newly 5 weeks spec	14.	71.6	{33., 115.}	411.429	0.656773	newly 5 weeks cumul	459.	1293.28	{632., 2185.}	181.76	0.404334	newly 51 weeks cumul	486.	1397.38	{686., 2344.}	187.528	0.412803
21	newly 3 weeks spec	38.	204.647	{95., 313.}	438.545	0.650969	newly 3 weeks cumul	270.	1159.82	{526., 1805.}	329.564	0.556017	newly 5 weeks spec	12.	66.1912	{27., 107.}	451.593	0.658751	newly 5 weeks cumul	303.	1343.	{628., 2114.}	343.234	0.569531	newly 52 weeks cumul	316.	1432.54	{661., 2278.}	353.337	0.579237
22	newly 3 weeks spec	162.	266.548	{123., 386.}	74.9502	0.19222	newly 3 weeks cumul	806.	1254.71	{577., 1826.}	67.87	0.180114	newly 5 weeks spec	58.	116.226	{52., 176.}	105.84	0.247092	newly 5 weeks cumul	951.	1549.65	{720., 2217.}	73.2065	0.188741	newly 48 weeks cumul	1042.	1785.94	{833., 2563.}	79.8898	0.20073
23	newly 3 weeks spec	22.	316.831	{150., 545.}	1340.14	1.07797	newly 3 weeks cumul	142.	1584.42	{709., 2757.}	1015.79	0.969008	newly 5 weeks spec	3.	129.442	{56., 237.}	4214.72	1.54478	newly 5 weeks cumul	156.	1921.32	{846., 3306.}	1131.62	1.01094	newly 49 weeks cumul	161.	2154.97	{968., 3665.}	1238.49	1.04646
24	newly 3 weeks spec	51.	235.194	{101., 409.}	361.165	0.585549	newly 3 weeks cumul	312.	1304.91	{538., 2250.}	318.241	0.543175	newly 5 weeks spec	15.	83.9104	{34., 169.}	459.801	0.657924	newly 5 weeks cumul	360.	1531.49	{639., 2673.}	325.415	0.549956	newly 51 weeks cumul	369.	1656.84	{685., 2884.}	349.007	0.573331
25	newly 3 weeks spec	160.	987.824	{429., 1802.}	517.39	0.723349	newly 3 weeks cumul	871.	4349.75	{1958., 8020.}	399.397	0.634759	newly 5 weeks spec	60.	482.662	{215., 915.}	704.436	0.836822	newly 5 weeks cumul	1024.	5526.22	{2501., 10 231.}	439.67	0.667559	newly 47 weeks cumul	1086.	6651.51	{3011., 12 332.}	512.478	0.721684
26	newly 3 weeks spec	96.	803.563	{421., 1325.}	737.045	0.867358	newly 3 weeks cumul	682.	3432.35	{1888., 5579.}	403.277	0.65028	newly 5 weeks spec	33.	411.563	{213., 627.}	1147.16	1.03953	newly 5 weeks cumul	784.	4412.92	{2420., 7195.}	462.872	0.698294	newly 45 weeks cumul	878.	5456.65	{2987., 8909.}	521.486	0.740665
27	newly 3 weeks spec	572.	458.836	{241., 820.}	37.2195	0.19658	newly 3 weeks cumul	2438.	1969.46	{1050., 3388.}	36.1809	0.190709	newly 5 weeks spec	284.	241.299	{137., 406.}	36.4253	0.184618	newly 5 weeks cumul	3101.	2540.64	{1371., 4349.}	36.1665	0.188516	newly 45 weeks cumul	3756.	3160.22	{1701., 5488.}	35.7768	0.182527
28	newly 3 weeks spec	8.	42.0448	{20., 77.}	425.56	0.656242	newly 3 weeks cumul	54.	261.194	{126., 486.}	383.693	0.622637	newly 5 weeks spec	1.	13.194	{5., 25.}	1219.4	1.0508	newly 5 weeks cumul	61.	297.015	{147., 542.}	386.91	0.627654	newly 52 weeks cumul	63.	313.433	{163., 562.}	397.512	0.637469
29	newly 3 weeks spec	378.	209.844	{109., 316.}	52.6455	0.334146	newly 3 weeks cumul	1806.	1022.3	{521., 1485.}	51.7312	0.32515	newly 5 weeks spec	145.	92.3594	{45., 142.}	48.6315	0.290234	newly 5 weeks cumul	2196.	1257.14	{635., 1859.}	51.4181	0.321524	newly 49 weeks cumul	2473.	1434.39	{738., 2146.}	50.9168	0.316029
30	newly 3 weeks spec	146.	200.532	{84., 393.}	60.9055	0.187122	newly 3 weeks cumul	831.	1151.57	{537., 2290.}	61.2062	0.185333	newly 5 weeks spec	47.	66.4416	{28., 123.}	64.189	0.192847	newly 5 weeks cumul	975.	1331.04	{625., 2653.}	60.1838	0.184816	newly 52 weeks cumul	1051.	1418.97	{669., 2829.}	59.3547	0.183984
31	newly 3 weeks spec	292.	984.516	{433., 1673.}	237.163	0.448622	newly 3 weeks cumul	1423.	3960.79	{1774., 6939.}	178.685	0.369471	newly 5 weeks spec	119.	584.516	{254., 1050.}	391.19	0.607769	newly 5 weeks cumul	1719.	5307.47	{2359., 9296.}	208.753	0.411764	newly 42 weeks cumul	1955.	7134.47	{3116., 12 677.}	264.934	0.482439
32	newly 3 weeks spec	532.	775.477	{170., 747.}	117.062	0.306803	newly 3 weeks cumul	2380.	3083.02	{797., 3541.}	96.8145	0.288503	newly 5 weeks spec	254.	391.046	{78., 374.}	131.617	0.329339	newly 5 weeks cumul	3005.	4036.14	{992., 4425.}	103.384	0.295399	newly 48 weeks cumul	3596.	4871.75	{1145., 5073.}	107.593	0.305284
33	newly 3 weeks spec	65.	22.1739	{10., 38.}	66.5106	0.536853	newly 3 weeks cumul	390.	131.623	{64., 231.}	66.8004	0.53037	newly 5 weeks spec	28.	6.56522	{2., 11.}	76.5528	0.714227	newly 5 weeks cumul	468.	150.304	{74., 260.}	68.2119	0.549619	newly 52 weeks cumul	508.	157.739	{77., 277.}	69.1886	0.563627
34	newly 3 weeks spec	107.	415.17	{170., 966.}	288.715	0.502785	newly 3 weeks cumul	548.	1941.32	{790., 4514.}	254.827	0.464561	newly 5 weeks spec	35.	190.245	{75., 452.}	443.558	0.635594	newly 5 weeks cumul	648.	2416.4	{992., 5663.}	273.227	0.48405	newly 48 weeks cumul	692.	2799.28	{1156., 6574.}	304.553	0.518266
35	newly 3 weeks spec	10.	94.5441	{34., 132.}	845.441	0.856272	newly 3 weeks cumul	81.	567.868	{217., 836.}	601.071	0.729871	newly 5 weeks spec	0.1	28.3382	{11., 42.}	28 238.2	2.31841	newly 5 weeks cumul	82.	649.279	{242., 958.}	691.804	0.782703	newly 52 weeks cumul	82.	683.897	{260., 1029.}	734.021	0.80534
36	newly 3 weeks spec	587.	841.09	{473., 1692.}	55.1044	0.151884	newly 3 weeks cumul	2638.	3748.49	{2098., 7428.}	53.9243	0.150394	newly 5 weeks spec	275.	402.806	{220., 786.}	58.209	0.158645	newly 5 weeks cumul	3306.	4733.03	{2633., 9386.}	54.6808	0.151276	newly 48 weeks cumul	3908.	5627.21	{3114., 11 136.}	55.235	0.151531
37	newly 3 weeks spec	138.	472.551	{214., 873.}	242.428	0.466452	newly 3 weeks cumul	721.	2259.9	{1106., 4115.}	213.439	0.431444	newly 5 weeks spec	53.	204.623	{98., 350.}	286.081	0.518572	newly 5 weeks cumul	854.	2774.3	{1367., 5003.}	224.86	0.446765	newly 49 weeks cumul	922.	3169.49	{1536., 5730.}	243.763	0.471369
38	newly 3 weeks spec	103.	445.652	{195., 821.}	332.672	0.573149	newly 3 weeks cumul	598.	1969.7	{875., 3661.}	229.381	0.455213	newly 5 weeks spec	29.	217.377	{102., 389.}	649.575	0.812456	newly 5 weeks cumul	693.	2496.94	{1129., 4645.}	260.309	0.494303	newly 46 weeks cumul	752.	3000.22	{1326., 5601.}	298.965	0.538356
39	newly 3 weeks spec	188.	372.19	{188., 588.}	100.439	0.248953	newly 3 weeks cumul	946.	1741.78	{858., 2695.}	87.9828	0.227045	newly 5 weeks spec	61.	162.921	{76., 291.}	167.239	0.3651	newly 5 weeks cumul	1112.	2152.21	{1060., 3419.}	96.293	0.242911	newly 48 weeks cumul	1224.	2478.59	{1238., 3933.}	104.257	0.258022
40	newly 3 weeks spec	395.	292.634	{109., 601.}	56.7588	0.321893	newly 3 weeks cumul	1868.	1415.45	{498., 2776.}	55.4461	0.308817	newly 5 weeks spec	218.	127.887	{41., 255.}	58.8836	0.393911	newly 5 weeks cumul	2391.	1737.28	{600., 3425.}	56.0117	0.322021	newly 49 weeks cumul	2892.	1982.06	{697., 3935.}	56.7929	0.339481
41	newly 3 weeks spec	12.	189.259	{77., 347.}	1477.16	1.10739	newly 3 weeks cumul	121.	1076.91	{460., 1986.}	790.011	0.860793	newly 5 weeks spec	2.	61.9138	{28., 120.}	2995.69	1.40238	newly 5 weeks cumul	135.	1246.93	{525., 2312.}	823.653	0.877634	newly 52 weeks cumul	137.	1331.53	{573., 2474.}	871.923	0.899541
42	newly 3 weeks spec	675.	999.682	{507., 1622.}	59.8406	0.165534	newly 3 weeks cumul	2966.	4031.68	{2067., 6277.}	51.4799	0.151756	newly 5 weeks spec	340.	574.015	{279., 922.}	76.1898	0.19464	newly 5 weeks cumul	3794.	5369.09	{2745., 8372.}	55.1341	0.157321	newly 43 weeks cumul	4690.	7142.27	{3608., 11 232.}	62.6362	0.169885
43	newly 3 weeks spec	545.	459.058	{217., 789.}	39.048	0.20096	newly 3 weeks cumul	2315.	2071.96	{1004., 3539.}	38.4086	0.18716	newly 5 weeks spec	245.	215.87	{102., 377.}	39.7752	0.19716	newly 5 weeks cumul	2918.	2604.28	{1245., 4533.}	38.5806	0.188293	newly 48 weeks cumul	3370.	3086.88	{1473., 5340.}	38.4325	0.183326
44	newly 3 weeks spec	215.	491.894	{265., 791.}	129.648	0.317805	newly 3 weeks cumul	1211.	2307.39	{1207., 3684.}	92.5356	0.244159	newly 5 weeks spec	93.	218.015	{110., 336.}	135.761	0.327598	newly 5 weeks cumul	1475.	2850.24	{1503., 4504.}	95.1351	0.249503	newly 48 weeks cumul	1690.	3294.58	{1721., 5244.}	96.8083	0.252834
45	newly 3 weeks spec	201.	470.046	{211., 935.}	135.492	0.307797	newly 3 weeks cumul	1070.	2331.18	{1072., 4411.}	120.582	0.284006	newly 5 weeks spec	90.	191.738	{85., 352.}	117.043	0.276595	newly 5 weeks cumul	1275.	2824.69	{1312., 5282.}	123.902	0.289638	newly 50 weeks cumul	1419.	3161.28	{1468., 5986.}	125.028	0.291582
46	newly 3 weeks spec	138.	75.0143	{23., 104.}	73.3437	0.487748	newly 3 weeks cumul	722.	432.329	{156., 614.}	71.5414	0.442277	newly 5 weeks spec	50.	24.9	{6., 41.}	71.4571	0.526506	newly 5 weeks cumul	861.	501.314	{176., 685.}	71.6509	0.45133	newly 52 weeks cumul	942.	534.043	{182., 731.}	71.8972	0.460489
47	newly 3 weeks spec	224.	429.957	{212., 692.}	95.3253	0.244379	newly 3 weeks cumul	1089.	1946.89	{971., 3146.}	84.085	0.223615	newly 5 weeks spec	77.	203.771	{87., 342.}	165.863	0.36622	newly 5 weeks cumul	1298.	2445.97	{1193., 3991.}	92.5622	0.239952	newly 47 weeks cumul	1446.	2901.39	{1388., 4751.}	103.874	0.262078
48	newly 3 weeks spec	86.	319.694	{148., 506.}	271.737	0.513397	newly 3 weeks cumul	493.	1624.63	{779., 2595.}	229.539	0.463097	newly 5 weeks spec	18.	132.339	{55., 222.}	635.215	0.805017	newly 5 weeks cumul	561.	1964.26	{919., 3148.}	250.135	0.488724	newly 50 weeks cumul	581.	2203.15	{1038., 3535.}	279.199	0.523219
49	newly 3 weeks spec	117.	148.382	{72., 269.}	52.1705	0.174078	newly 3 weeks cumul	638.	719.434	{333., 1280.}	47.2261	0.175909	newly 5 weeks spec	40.	65.9737	{32., 131.}	75.8553	0.200068	newly 5 weeks cumul	753.	883.342	{411., 1608.}	48.4798	0.172758	newly 48 weeks cumul	828.	1010.14	{464., 1857.}	50.0461	0.171351
50	newly 3 weeks spec	261.	367.826	{151., 568.}	66.6611	0.191843	newly 3 weeks cumul	1311.	1761.74	{762., 2766.}	62.551	0.189389	newly 5 weeks spec	104.	161.	{60., 253.}	77.7174	0.213108	newly 5 weeks cumul	1579.	2167.2	{916., 3394.}	64.1261	0.190157	newly 49 weeks cumul	1754.	2477.9	{1038., 3888.}	66.3138	0.191201
